# Effect of MAO-B Inhibitors on Neurometabolic Profile of Patients Affected by Parkinson Disease: A Proton Magnetic Resonance Spectroscopy Study

**DOI:** 10.3390/jcm11071931

**Published:** 2022-03-30

**Authors:** Lilla Bonanno, Rosella Ciurleo, Silvia Marino, Claudio Ruvolo, Rosa Morabito, Alessia Bramanti, Francesco Corallo

**Affiliations:** 1 Neuroimaging Laboratory, IRCCS Centro Neurolesi Bonino-Pulejo, 98124 Messina, Italy; lilla.bonanno@irccsme.it (L.B.); silvia.marino@irccsme.it (S.M.); claudio.ruvolo@irccsme.it (C.R.); rosa.morabito@irccsme.it (R.M.); francesco.corallo@irccsme.it (F.C.); 2 Department of Medicine Surgery and Dentistry ‘Salerno Medical School’, University of Salerno, 84084 Fisciano, Italy; alessia.bramanti@gmail.com

**Keywords:** cerebral cortex, choline, disease progression, MAO-B inhibitors, N-acetylaspartate, neurometabolites, Parkinson’s disease, proton magnetic resonance spectroscopy, rasagiline, selegiline

## Abstract

Parkinson’s Disease (PD) is the most common neurodegenerative movement disorder whose treatment is symptomatic. No suitable methods for assessing the effects of dopaminergic drugs on disease progression in clinical trials have yet been provided. The aim of this longitudinal study is to evaluate the influence of rasagiline and selegiline on neurometabolic profile in de novo PD patients by using Proton Magnetic Resonance Spectroscopy (^1^H-MRS). We enrolled de novo PD patients who were divided into two groups of 20 patients each, according to the dopaminergic treatment prescribed at the baseline visit (rasagiline or selegiline). At the baseline visit and after 12 months, all patients underwent neurological evaluation as well as ^1^H-MRS. Forty healthy controls (HC) underwent ^1^H-MRS at baseline and after 12 months. PD patients, compared to HC, showed significantly lower concentrations of NAA in the motor cortex, while the Cho levels showed a decreasing trend. After 12 months of therapy, the ^1^H-MRS study revealed that rasagiline and selegiline in a similar way were able to restore the NAA levels to values similar to those of HC. In addition, this neurometabolic change showed a correlation with UPDRS-III scores. This is the first longitudinal study that provides preliminary evidence that ^1^H-MRS may be a suitable method to evaluate objectively the influence of MAO-B inhibitors on the neurometabolic profile of PD patients. These results could open a new scenario on the hypothesis of a drug-induced slowing effect of PD progression.

## 1. Introduction

Parkinson’s Disease (PD) is the most common neurodegenerative movement disorder. PD is characterized by progressive degeneration of dopaminergic neurons in substantia nigra pars compacta (SNc). This loss of dopaminergic neurons results in decreased levels of dopamine in the putamen of the dorsolateral striatum, leading to dysfunction of direct and indirect pathways of movement control that involve cortico-basal-thalamo-cortical loops [1]. Currently, PD treatment is symptomatic. One of the major challenges in PD is the development of disease-modifying therapies, such as neuroprotective or cell-based restorative agents. So far, the main clinical outcome measure used in clinical trials to detect disease progression has been the motor part of the Unified Parkinson’s Disease Rating Scale (UPDRS-III), which does not allow for distinctions between symptomatic and disease-modifying drug effects. Therefore, there is an unmet need to use an objective biomarker to evaluate disease progression and the effects of dopaminergic drugs.

Magnetic Resonance Spectroscopy (MRS) is a non-invasive imaging technique for exploration in vivo of intracellular metabolic status and may provide a neuroimaging biomarker of normal biological and pathological processes or response to therapeutic intervention in PD. In vivo Proton MRS (^1^H-MRS)is a useful tool for the detection of neurometabolic changes in patients with PD [2,3]. The metabolites detectable and quantifiable with ^1^H-MRS include (i) N-acetylaspartate (NAA), interpreted as a marker of neuronal function; (ii) choline-containing compounds (Cho), considered as a marker of membrane turnover or inflammation; (iii) creatine + phosphocreatine (Cr), which reflects energy metabolites; (iv) myo-inositol (mI), suggested as a glial marker and (v) glutamate with glutamine (Glu), which is the main excitatory neurotransmitter in the brain [4].

As suggested by other studies [5,6,7,8,9,10,11,12,13,14,15,16,17,18], the concentrations of the main neurometabolites detectable by ^1^H-MRS in cortico-basal ganglia networks are different between PD patients and healthy control subjects. To date, few ^1^H-MRS studies on possible brain metabolic changes induced by dopaminergic therapy in PD patients have been reported [6,19,20,21]. In particular, these short-term studies, which investigated the influence of levodopa, ropinirole and pergolide on the neurometabolic profile of PD patients, have suggested that dopaminergic therapy may partially re-establish normal neurometabolite levels as a result of drug-induced restoration of the normal pattern of cortico-basal-thalamo-cortical functions. To our knowledge, there are no ^1^H-MRS studies that have evaluated the influence of MAO-B inhibitors, such as rasagiline and selegiline, on the neurometabolic profile of PD patients.

A critical node of the cortico-basal-thalamo-cortical neural network impacted by PD is the motor cortex, which plays a key role in generating neural impulses that regulate movements. The functional activity of the motor cortex is altered in PD and related to many of the motor signs. Although the functional activity of the motor cortex can be partially restored with acute dopaminergic medication, as demonstrated by improvement of motor performance, it continues to deteriorate with disease progression [22].

The aim of this study was to assess the effects of rasagiline and selegiline on neurometabolic profile in the motor cortex of de novo PD patients by using ^1^H-MRS in order to understand if potentially neuroprotective drugs could slow down the progressive alteration of functional activity of motor cortex as a result of partial restoration of neurometabolites. To verify this, we aimed to evaluate the correlation between the neurometabolic changes and clinical motor outcomes, as revealed by UPDRS-III scores.

## 2. Materials and Methods

### 2.1. Participants

Twenty de novo PD patients, who were going to undertake dopaminergic treatment with selegiline and 20 with rasagiline, and 40 age- and sex-matched healthy controls (HC) were recruited from IRCCS Centro Neurolesi Bonino-Pulejo of Messina, Italy. Drug-treated groups of PD patients were matched for age, gender and disease duration. For patients, inclusion criteria were: (1) male or female patients aged between 45 and 60 years old; (2) clinical diagnosis of PD according to UK Brain Bank Criteria; (3) beginning of the treatment with rasagiline or selegiline at study entry as part of their routine clinical care and according to the approved label. Exclusion criteria were: (1) absolute contraindications to MRI; (2) concomitant neurological disease; (3) any medication that could interact with brain neurotransmitters (i.e., antidepressants, antipsychotics); (4) inability to provide informed consent.

### 2.2. Ethics

All subjects gave written informed consent for participation in the study in accordance with the Declaration of Helsinki. IRCCS Centro Neurolesi Bonino-Pulejo Ethical Committee, Messina, Italy, approved the study protocol (approval No.6/2016) in December 2016.

### 2.3. Symptoms Evaluation/Outcome Measures

At the baseline visit, after signing the informed consent, demographics, most affected side, medical history and current therapies were collected from each enrolled PD patient. PD patients underwent an extensive neurological examination in which disease stage and severity were rated by Hoehn–Yahr stage (H&Y) [23] and UPDRS (parts 1–4) [24]. In addition, all the eligible PD patients, according to the inclusion and exclusion criteria, underwent MRI and ^1^H-MRS examinations by using an MR Scanner operating at 3T at entry study, before the beginning of the MAO-B inhibitor treatment, and after 12 months. Each PD patient was assessed with UPDRS-III after 12 months of therapy with selegiline (10 mg/die) or rasagiline (1 mg/die) in the ON period. The HC underwent MRI/^1^H-MRS examinations at baseline visit and after 12 months.^1^H-MRS data analysis was conducted in a rater-blinded manner by the study investigator that was not otherwise involved in the therapeutic and clinical management of patients.

### 2.4. MRI and ^1^H-MRS Acquisition

**Figure 1:**
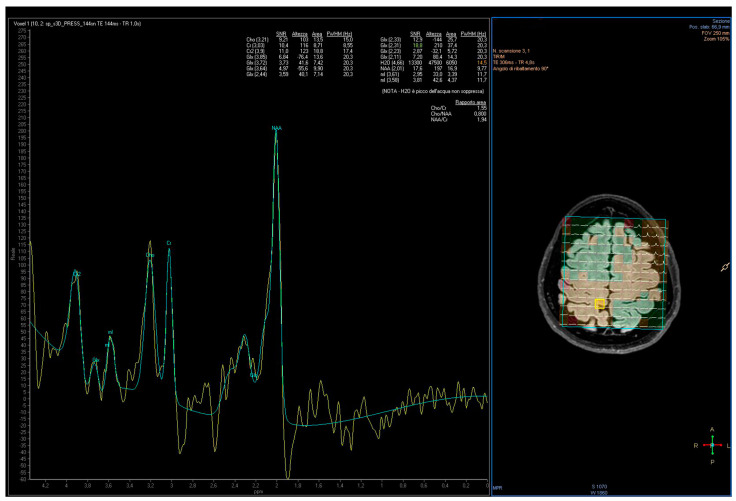
Voxel position from the motor cortex and representative spectra obtained from a PD patient. Lengend: Commas represents the decimal separator.

All subjects were examined by using an MRI protocol which included combined conventional MRI and ^1^H-MRS examinations of the brain. The MRI acquisition was performed by 3T whole-body MRI equipment (Achieva, Philips Medical System, Best, The Netherlands), using a 32-element phased array sensitivity-encoding (SENSE) head coil. The MR system was equipped with gradients achieving a maximum slew rate of 200 mT/m/ms and maximum strength of 80 mT/m. The 3T imaging protocol included 3D T1-weighted Fast Field Echo (FFE), 3D FLAIR, two-dimensional coronal T2-weighted Fast Spin Echo (FSE). Three-dimensional-T1-weighted FFE images were acquired with the following parameters: Repetition Time (TR) 8.2 ms; Echo Time (TE) 3.7 ms; section thickness 1 mm; number of signals averaged 1 and reconstruction matrix 512 × 512. 3D-FLAIR images were acquired with TR 12,000 ms, TE 140 ms and T2-weighted. FSE images were acquired with TR 4100 ms, TE 100 ms, a section of thickness 2–3 mm, number of signals averaged to 1 and a reconstruction matrix of 512 × 512. The MR images were used to select intracranial volumes of interest (VOIs) for spectroscopy. For the MRS section, 2D-MRS sequence, point-resolved spectroscopy (PRESS), was used (TE: 35 ms, TR: 2000 ms, average 128). The VOI of multi-voxel MRS sections was 180 mm × 180 mm × 10 mm, thus the volume of one voxel was (180/16)^2^ × 10=1.3 cm^3^. Corresponding unsuppressed water spectra with equal TE and TR were additionally acquired. Before data collection, the automatic shim provided by the manufacturer was carried out to optimize field homogeneity. The multi-voxel section encompassed the motor cortex in order to include both hemispheres (Figure 1).

### 2.5. Data Processing

The resonance intensity values of metabolites were assessed using LCModel/LCMgui package (Version 6.3—Steven Provencher, Oakville, ON, Canada).

The concentrations of the neurometabolites were estimated by fitting the spectrum to a linear combination of “basic spectra” of each neurometabolite, provided by LCModel software for a 3T PRESS acquisition with a TE = 35 ms. Using this software, Gaussian-fitted peak areas were determined relative to a baseline computed from a moving average of the noise regions of each spectrum. The main neurometabolites detected were NAA peak at 2.0 ppm, Cr peak at 3.0 ppm and Cho peak at 3.2 ppm.

Cramer-Rao Lower Bounds (given as % of the Standard Deviation (SD) value by the LCModel software) were used as a criterion for the rejection of poor-quality spectra. Only spectra with a percentage of SD ˂ 20 were considered in the study. For data analysis, the concentration of neurometabolites expressed as the ratio of NAA and Cho to Cr in the motor cortex was used.

### 2.6. Statistical Analysis

Description of groups was reported for demographic and clinical variables. Continuous variables were expressed as mean ± standard deviation, whereas categorical variables in frequencies and percentages. No parametric analysis was carried out because the results of the Shapiro normality test indicated that most of the target variables were not normally distributed. The numerical data were presented as median, and first-third quartile in nonnormal distribution and the χ^2^ test and the Mann–Whitney U test was used for inter-group analysis, when appropriate. The Wilcoxon signed-rank test was used in order to compare the clinical variables (UPDRS-III) and metabolite ratios at baseline (T0) and after 12 months (T1). We performed an interaction effect analysis (improved time) by calculating the T1–T0 differences in clinical variable scores. The correlations between UPDRS-III and metabolic ratios in each patient group were performed by Spearman correlation analysis. Analyses were performed using an open-source R3.0 software package (R Foundation for Statistical Computer, Vienna, Austria). A 95% of confidence level was set with a 5% alpha error. Statistical significance was set at *p* < 0.05.

## 3. Results

### 3.1. Demographic and Clinical Data

Overall, in this study 40 de novo PD patients and 40 HC were enrolled. No significant differences between PD patients and HC in age (*p* = 0.76) [PD patients: 52.2 ± 5.80; HC: 52.6 ± 5.27] and sex (χ^2^ = 0.83; *p* = 0.36) [PD patients: males = 22, females = 18; HC: males = 26, females = 14) were found. The patients displayed a mean value of 4.68 ± 0.69 for UPDRS-I, 5.20 ± 0.69 for UPDRS-II and 18.2 ± 2.19 for UPDRS-III.

### 3.2. H-MRS Data at Baseline

The inter-group analysis highlighted the differences in ^1^H-MRI data at baseline (T0) between PD patients and HC (Table 1). In particular, the Mann–Whitney U test indicated that in the motor cortex the NAA/Cr ratio was significantly lower for PD patients than for the HC (*p* < 0.0001) and the Cho/Cr ratio showed a decreasing trend for PD patients compared to HC (*p* = 0.06). In the motor cortex of the HC, no significant differences in metabolite levels after 12 months from baseline were observed (*p* > 0.05).

### 3.3. Dopaminergic Treatment Patient Group

The 40 PD patients enrolled were divided into two groups according to the dopaminergic treatment prescribed at the baseline visit: rasagiline and selegiline treatment group. The groups, at baseline and after 12 months, are homogeneous for ^1^H-MRI data and clinical assessment (Table 2).

### 3.4. Rasagiline Treatment Patient Group

**Figure 2:**
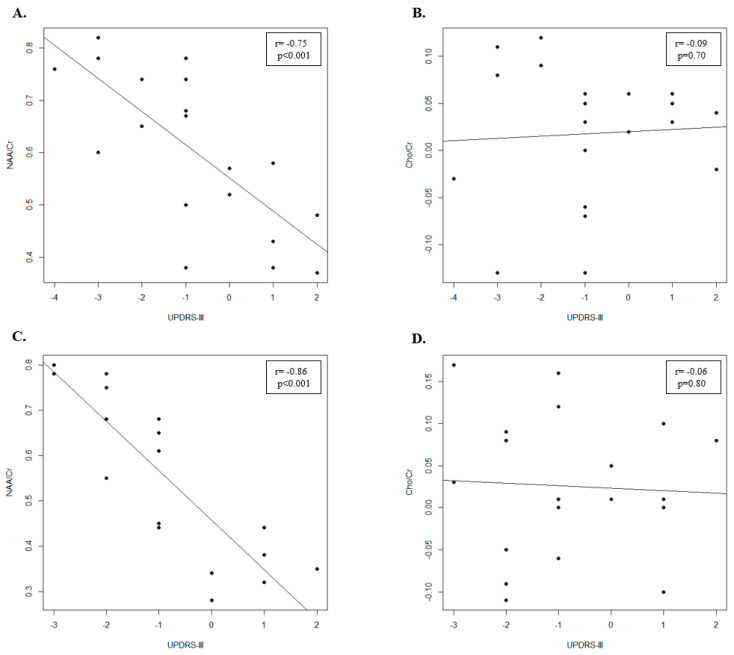
Scatter plot of UPDRS-III and the metabolite ratios after 12 months of therapy in PD patients. (**A**) Scatter plot of UPDRS-III and NAA/Cr metabolite ratio in Rasagiline treatment group. (**B**) Scatter plot of UPDRS-III and Cho/Cr metabolite ratio in Rasagiline treatment group. (**C**) Scatter plot of UPDRS-III and NAA/Cr metabolite ratio in Selegiline treatment group. (**D**) Scatter plot of UPDRS-III and Cho/Cr metabolite ratio in Selegiline treatment group.

In the rasagiline group (Table 2), the NAA/Cr and Cho/Cr ratios increased in the motor cortex after 12 months of therapy. In particular, the NAA/Cr ratio increased significantly (*p* < 0.001), while the Cho/Cr ratio increases but not significantly (*p* = 0.25). Clinical assessment showed that significant differences in UPDRS-III (*p* = 0.05) between T0 and T1 exist (Table 2). Moreover, we found a significant negative correlation between UPDRS-III score and NAA/Cr ratio (r = −0.75; *p* < 0.001) (Figure 2A) and no significant negative correlation between UPDRS-III score and Cho/Cr ratio (r = −0.09; *p* = 0.70) (Figure 2B).

### 3.5. Selegiline Treatment Patient Group

In the selegiline group (Table 2), the NAA/Cr and Cho/Cr ratios increased in the motor cortex after 12 months of therapy. However, while the NAA/Cr ratio increased significantly (*p* < 0.001) the increase of Cho/Cr ratio was not significant (*p* = 0.20). In addition, we found that significant differences in UPDRS-III (*p* = 0.03) (Table 2) between T0 and T1 exist. Finally, we showed a significant negative correlation between UPDRS-III score and NAA/Cr ratio (r = −0.86; *p* < 0.001) (Figure 2C) and a negative correlation between UPDRS-III score and Cho/Cr ratio, even if it was not significant (r = −0.09; *p* = 0.70) (Figure 2D).

## 4. Discussion

In this longitudinal ^1^H-MRS study, we assessed the neurometabolic changes in de novo PD patients after 12 months of therapy with rasagiline and selegiline.

Overall, before the beginning of therapy, de novo PD patients showed significantly lower concentrations of NAA compared to HC in the motor cortex. The Cho concentrations showed a trend towards significance for decrease.

Some of our findings are in agreement with the previous studies. Indeed, lower levels of NAA have been reported in the motor cortex [7] and prefrontal lobe [16,18]. While reduced levels of NAA reflect the decrease of neuron density and then, the diffuse neuronal degeneration in PD, the reduced levels of Cho make it difficult to understand their role in the pathophysiology of PD. In general, an increase in Cho concentration is the result of intensified membrane turnover, which is how it occurs in inflammatory processes. Here, it may be that a decrease in the Cho levels is due to a reduced membrane turnover, perhaps as a result of cell loss.

To our knowledge, a few ^1^H-MRS studies investigated the effects of the therapy on the neuro-metabolic profile of PD patients. A ^1^H-MRS study [19] showed an increase in Cho/Cr and NAA/Cr ratios in the motor cortex of de novo PD patients and an improvement in motor performance 6 months after pergolide treatment. In the same way, we have studied 20 de novo drug-naïve PD patients before and after therapy with ropinirole by using multi-voxel 1H-MRSI at 1.5 Tesla [20]. After 10 months of therapy with ropinirole, PD patients showed a significant increase in NAA levels from the motor cortex and a highly significant clinical improvement, as shown by the UPDRS motor sub-score. No changes in Cho/Cr were found. Another study using ^1^H-MRS at 3 T to explore the metabolic profile in the putamen of PD patients has found that in those patients who had taken L-DOPA therapy (drug-on), the NAA and Cr concentrations increased back to near control levels, providing indirect evidence of an effect of acute administration of L-DOPA in increasing neuronal function.

In this study, the influence of the MAO-B inhibitors on the metabolite profile of the cerebral cortex has been investigated by high-field ^1^H-MRS.

After 12 months of therapy, NAA levels increased equally in the motor cortex of both selegiline- and rasagiline-treated patients. No significant change in Cho concentration in PD patients treated with rasagiline and selegiline was found. In addition, 12 months after treatment with rasagiline and selegiline, PD patients showed a clinical improvement in motor performance, as demonstrated by UPDRS motor sub-scores.

Studies in vitro and with experimental models have shown that rasagiline and selegiline have neuroprotective effects, although clinical studies have not completely confirmed these effects due to the lack of objective biomarkers to quantify disease progression in PD. In particular, it has been reported that selegiline enhances the synthesis of nerve growth factors, protects dopaminergic neurons from glutamate-mediated neurotoxicity and from other toxic factors, reduces the production of oxidative radicals, up-regulates superoxide dismutase and catalase, and has anti-apoptotic effects, independently of MAOB inhibition [25,26]. Recently, rasagiline has been suggested to induce neuroprotective effects similar to those of selegiline [27,28,29].

In our study, we showed that rasagiline and selegiline in a similar way, seem to restore NAA levels. Therefore, it could be hypothesized that these drugs are able to reduce neuronal degeneration in PD. The recovery of NAA concentrations to near HC could be the upstream effect of the ability of MAO-B inhibitors to restore, also partially, the neuron functions by neuroprotective mechanisms. NAA is synthesized in the neuronal mitochondria by the action of the L-aspartate N-acetyltransferase via a reaction that utilizes L-aspartate and acetyl coenzyme A. MAO-B inhibitors seem to be able to protect mitochondria against pro-oxidant-induced damage [30] and this could reflect an increase of NAA synthesis at the level of the neuronal mitochondria.

The recovery of NAA concentrations to near the HC could be an upstream effect of the ability of MAO-B inhibitors to modulate the functional cortical activity, underlying motor PD symptoms, perhaps by neuroprotective mechanisms.

Indeed, we found a negative correlation between the UPDRS-III scale scores and NAA concentrations. No correlation was found between the UPDRS-III scale scores and Cho concentration in the motor cortex of patients treated with both MAO-B inhibitors. Although the changes in UPDRS scores cannot be directly related to the effect of any potential neuroprotective agent, their correlation with NAA level changes could help to separate symptomatic effects from a realistic effect on disease progression exerted by this therapy. Therefore, these results could support that ^1^H-MRS is a noninvasive, valid and objective biomarker to evaluate the effects of MAO-B inhibitors on the decrease of neuronal degeneration in PD, and then on the slowing of disease progression.

However, the present study has some limitations. First, the sample size was limited, as we had some drop-out at the follow-up evaluation. Eighteen of all patients initially enrolled in our study were excluded at the follow-up visit because of poor-quality spectra. In addition, to support our hypothesis it would have been crucial to have a group of PD patients untreated and followed up at the same time. However, it is difficult to enroll untreated PD patients in studies because of ethical implications.

Biological fluctuations of neurometabolite concentrations over time, partly because of aging, remain as potential confounders, although the HC showed no differences in metabolite levels between ^1^H-MRS performed at baseline and after 12 months. Further ^1^H-MRS investigations of other structures, such as basal ganglia, and the detection of absolute quantification of neurometabolites, rather than their ratio to the signal of Cr and a larger sample, would be useful to confirm our results.

In conclusion, this ^1^H-MRS study seems to confirm, in an objective and non invasive way, that in de novo PD patients there is an alteration of neurometabolite levels compared with HC. In addition, ^1^H-MRS is a valid instrumental method to detect the potential restoration of NAA concentration after therapy with MAO-B inhibitors as a result of their likely ability to influence disease progression. This is the first longitudinal study that provides preliminary evidence that ^1^H-MRS may be a valid biomarker to be used in clinical trials in order to detect the effects of disease-modifying dopaminergic drugs.

## Figures and Tables

**Table 1:** Demographic and imaging characteristics of de novo PD patients and HC groups.

		Parkinson Disease	Health Control	*p*
**Demographic characteristics**				
N. Sample		40	40	
Age (Mean ± SD)		52.2 ± 5.80	52.6 ± 5.27	0.76
Gender				
	Male	22 (55%)	26 (65%)	0.36
	Female	18 (45%)	14 (35%)	
**^1^H-MRI Data**				
NAA/Cr (Median) (I–III quartile)	T0	2.29 (2.15–2.45)	2.85 (2.81–2.89)	<0.0001 *
	T1	2.86 (2.83–2.9)	2.85 (2.81–2.89)	0.24
	*p*	<0.0001 *	-	
Cho/Cr (Median) (I–III quartile)	T0	0.4 (0.38–0.43)	0.41 (0.39–0.45)	0.06
	T1	0.41 (0.39–0.44)	0.41 (0.39–0.45)	0.65
	*p*	0.03 *	-	

**Legend:** Cho = Choline; Cr = Creatine; HC = Health Control; NAA = N-acetylaspartate; PD = Parkinson Disease; SD = Standard Deviation. * *p* < 0.05.

**Table 2:** Demographic and clinical and imaging characteristics of Rasagiline and Selegiline treatment patient group.

		Rasagiline	Selegiline	*p*
**Demographic characteristics**				
N. Sample		20	20	
Age (Mean ± SD)		52.5 ± 6.23	52.8 ± 5.48	0.73
Gender				
	Male	9 (45%)	13 (65%)	0.20
	Female	11 (55%)	7 (35%)	
**Clinical Assessment**				
UPDRS-III	T0	18.0 (16.7–20.0)	18.5 (16.0–20.0)	0.75
	T1	17.0 (15.0–20.0)	17.5 (16.0–19.2)	0.70
	*p*	0.05 *	0.03 *	
**^1^H-MRI Data**				
NAA/Cr (Median) (I–III quartile)	T0	2.27 (2.16–2.36)	2.36 (2.14–2.47)	0.14
	T1	2.86 (2.83–2.90)	2.87 (2.84–2.9)	0.49
	*p*	<0.001 *	<0.001 *	
Cho/Cr (Median) (I–III quartile)	T0	0.40 (0.38–0.42)	0.40 (0.34–0.44)	0.84
	T1	0.44 (0.38–0.46)	0.41 (0.38–0.45)	0.54
	*p*	0.25	0.20	

**Legend.** Cho = Choline; Cr = Creatine; HC = Health Control; NAA = N-acetylaspartate; PD = Parkinson Disease; SD = Standard Deviation. * *p* < 0.05.

## Data Availability

The data presented in this study are available on request from the corresponding author. The data are not publicly available due to ethical.
